# CB_1_ receptor-dependent and -independent inhibition of excitatory postsynaptic currents in the hippocampus by WIN 55,212-2

**DOI:** 10.1016/j.neuropharm.2007.07.003

**Published:** 2008-01

**Authors:** Beáta Németh, Catherine Ledent, Tamás F. Freund, Norbert Hájos

**Affiliations:** a Institute of Experimental Medicine, Hungarian Academy of Sciences, Szigony u. 43, Budapest H-1450, Hungary; b Institut de Recherche Interdisciplinaire en Biologie Humaine et Moleculaire, Universite Libre de Bruxelles, 1070 Brussels, Belgium

**Keywords:** Brain slices, Glutamate, Transmitter release, Hippocampus, Pyramidal cell, Cannabinoids

## Abstract

We investigated the effect of a synthetic cannabinoid, WIN 55,212-2 on excitatory postsynaptic currents (EPSCs) evoked by stimulation of Schaffer collaterals in CA1 pyramidal cells. Bath application of WIN 55,212-2 reduced the amplitude of EPSCs in dose-dependent manner tested between 0.01 nM and 30 μM. In rats and mice, this cannabinoid ligand inhibited excitatory synapses in two steps at the nM and μM concentrations. When the function of CB_1_ cannabinoid receptors (CB_1_R) was impaired, either by the application of a CB_1_R antagonist AM251, or by using CB_1_R knockout mice, WIN 55,212-2 in μM concentrations could still significantly reduced the amplitude of EPSCs. WIN 55,212-2 likely affected the efficacy of excitatory transmission only at presynaptic sites, since both at low and high doses the paired pulse ratio of EPSC amplitude was significantly increased. The inactive enantiomer, WIN 55,212-3, mimicked the effect of WIN 55,212-2 applied in high doses. In further experiments we found that the CB_1_R-independent effect of 10 μM WIN 55,212-2 at glutamatergic synapses was fully abolished, when slices were pre-treated with ω-conotoxin GVIA, but not with ω-agatoxin IVA.

These data suggest that, in the hippocampus, WIN 55,212-2 reduces glutamate release from Schaffer collaterals solely via CB_1_Rs in the nM concentration range, whereas in μM concentrations, WIN 55,212-2 suppresses excitatory transmission, in addition to activation of CB_1_Rs, by directly blocking N-type voltage-gated Ca^2+^ channels independent of CB_1_Rs.

## 1 Introduction

The type 1 cannabinoid receptors (CB_1_Rs) have been shown to control the release of different neurotransmitters, but the mechanisms underlying the regulation of synaptic communication could substantially vary between brain regions (Freund et al., 2003). Pharmacological results, suggesting a presynaptic locus of action of cannabinoid receptor ligands, have been fully supported by immunohistochemical data. Several studies demonstrated at the electron microscopic level that CB_1_Rs decorated both inhibitory and excitatory axon terminals (Katona et al., 1999, 2006; Kawamura et al., 2006). In addition, recent high-resolution quantitative studies established that CB_1_Rs were found all around the axon membrane, but were enriched in the perisynaptic annulus and on preterminal segments, whereas immunolabelling was weaker in the synaptic active zone (Nyiri et al., 2005; Kawamura et al., 2006). This subcellular distribution of CB_1_Rs might imply an action on several regulatory mechanisms of transmitter release, including the control of Ca^2+^ entry via voltage-dependent Ca^2+^ channels (primarily by receptors located in the perisynaptic annulus), the reduction of axonal conduction (by receptors present on the preterminal segments), or a direct action on exocytosis (Wilson et al., 2001; Diana and Marty, 2003).

In spite of the direct anatomical evidence, several pharmacological observations suggest that some synthetic cannabinoid agonists (mainly WIN 55,212-2) could also have a CB_1_R-independent action on synaptic glutamate release. This possibility has been fuelled primarily by experiments using CB_1_R knockout mice. Our laboratory was the first to show that, in the absence of CB_1_Rs, WIN 55,212-2 was still able to reduce excitatory, but not inhibitory postsynaptic currents in CA1 pyramidal neurons (Hájos et al., 2001). Moreover, WIN 55,212-2 was more potent in suppressing GABAergic than glutamatergic transmission (Hoffman and Lupica, 2000; Ohno-Shosaku et al., 2002; Hájos and Freund, 2002), providing further support for the possible presence of CB_1_R-independent binding site at excitatory synapses. Importantly, AM251, a CB_1_R antagonist prevented the reduction of synaptic inhibition after application of WIN 55,212-2, whereas glutamatergic transmission could still be suppressed by about 50% in the presence of AM251 (Hájos and Freund, 2002). In contrast to the above findings showing that hippocampal glutamatergic synapses were effectively regulated independent of CB_1_Rs, electrophysiological data from other groups suggested that CB_1_Rs were solely responsible for the cannabinoid modulation of excitatory synaptic transmission in the hippocampus (Ohno-Shosaku et al., 2002; Domenici et al., 2006; Takahashi and Castillo, 2006).

To shed light on the reasons behind the contradictory findings regarding the involvement of CB_1_R-dependent vs. -independent mechanisms in the regulation of hippocampal excitatory synapses, we re-examined the effect of WIN 55,212-2 on monosynaptically evoked excitatory postsynaptic currents (EPSCs) in CA1 pyramidal cells. All these experiments were performed in a modified submerged recording conditions (Hájos et al., 2005).

## 2 Methods

Experiments were carried out according to the guidelines of the institutional ethical code and the Hungarian Act of Animal Care and Experimentation (1998. XXVIII. section 243/1998.). Male Wistar rats (14–18 days old), as well as wild type and CB_1_R knockout mice (15–25 days old, CD1 strain) were used. The animals were deeply anaesthetized with isoflurane followed by decapitation. After opening the skull, the brain was quickly removed and immersed into ice-cold cutting solution containing (in mM: sucrose 252; KCl 2.5; NaHCO_3_ 26; CaCl_2_ 0.5; MgCl_2_ 5; NaH_2_PO_4_ 1.25; glucose 10). The solution had been bubbled with 95% O_2_/5% CO_2_ (carbogen gas) for at least 30 min before use. Thick horizontal slices (350 μm from mice and 400 μm from rats) were prepared using a Leica VT1000S Vibratome. The CA3 region was removed to prevent epileptic burst firings. The slices were stored in an interface type chamber containing ACSF (in mM: 126 NaCl, 2.5 KCl, 26 NaHCO_3_, 2 CaCl_2_, 2 MgCl_2_, 1.25 NaH_2_PO_4_, and 10 glucose) at room temperature for at least 1 h before recording. After the initial incubation period, slices were transferred individually into a submerged type recording chamber.

Whole-cell patch-clamp recordings were obtained at 30–32 °C from CA1 pyramidal cells visualized by infrared DIC videomicroscopy (Zeiss Axioscope, Germany). Patch electrodes were pulled from borosilicate glass capillaries with an inner filament (1.5 mm O.D.; 1.12 mm I.D., Hilgenberg, Germany) using a Sutter P-87 puller. Electrodes (∼3–6 MΩ) were filled with a solution containing (in mM) 80 CsCl, 60 Cs-gluconate, 3 NaCl, 1 MgCl_2_, 10 HEPES, 2 Mg-ATP, and 5 QX-314 (pH 7.2–7.3 adjusted with CsOH; osmolarity 275–290 mOsm). Excitatory postsynaptic currents (EPSCs) were recorded at a holding potential of −65 mV. Slices were perfused with ACSF containing 70–100 μM picrotoxin to block inhibitory neurotransmission. The solution was bubbled with carbogen gas at room temperature and perfused at a flow rate of 3–4.5 ml/min in a slice chamber optimized for laminar flow to ensure the stability of the amplitude of evoked currents and a better oxygenation of submerged slices (Hájos et al., 2005). To evoke EPSCs, the stimulating electrode was placed in the stratum radiatum of CA1. Pairs of electrical stimuli separated by 50 ms were delivered via a theta glass pipette (Sutter Instrument Company, Novato, CA) filled with ACSF at 0.1 Hz using a Supertech timer and isolator (Supertech LTD, Pécs, Hungary, http://www.superte.ch). Access resistances (between 4 and 18 MΩ, compensated 65–70%) were frequently monitored and remained constant (±20%) during the period of analysis. Signals were recorded with a Multiclamp 700A (Molecular Devices, Sunnyvale, CA), filtered at 2 kHz, digitized at 6 kHz (National Instruments PCI-6024E A/D board, Austin, TX), and analyzed off-line with the EVAN program (courtesy of Prof. I. Mody, UCLA, CA).

The drug was perfused in a given concentration until the maximal effect was reached. The time needed for maximal inhibition was usually 6–8 min. To avoid the possible effect of a changing pH, we added the same amount of HCl to the control solution. The concentration response relationship for WIN 55,212-2 was obtained as follows: control EPSC amplitudes in a 2–3 min time window were compared to those measured after 10 min drug application for the same period of time. Only those experiments were included that had stable amplitudes at least for 10 min before drug application. After each experiment, the tubing made of Teflon was washed with ethanol for 10 min and with ACSF for 15 min. Each data point represents the mean ± SEM of the maximal inhibition of the evoked EPSCs (*n* = 3–7). EC_50_ values were estimated by fitting a curve to the points of the dose response plots obtained in rats or wild type mice using the equation of *f*(*x*) = *a*/(1 + exp(−(*x* − *c*)/*b*)) + (100 − *a*)/(1 + exp(−(*x* − *e*)/*d*)), where ‘*c*’ and ‘*e*’ give the values for high and low affinity binding sites, respectively. The data points obtained in the presence of AM251 or in CB1 knockout mice were fitted by the equation of *f*(*x*) = *a*/(1 + exp(−(*x* − *c*)/*b*)), where ‘*c*’ gives the value of EC_50_. The curve fitting was done using Origin 7.5 (OriginLab Corporation, MA). The paired pulse ratio was calculated from the mean amplitude of the second EPSCs divided by the mean amplitude of the first EPSCs. The paired pulse ratio after drug treatment was compared with the control using Wilcoxon matched pairs test in Statistica 6.1 (Statsoft, Inc., Tulsa, OK). Data are presented as mean ± SEM.

Picrotoxin, WIN 55,212-2 and WIN 55,212-3 were purchased from Sigma-Aldrich, AM251 was obtained from Tocris, while ω-conotoxin GVIA and ω-agatonix IVA from Alomone Labs. For all experiments, WIN-55,212-2 was dissolved in 0.1N HCl giving a 20 mM stock solution stored at 4 °C. AM251 was dissolved in DMSO (100 mM) and stored at −20 °C. WIN 55,212-3 dissolved in DMSO (100 mM) was stored at 4 °C. From these stock solutions, the final dilution of drugs was done in ACSF containing picrotoxin under constant stirring and the prepared solution was bath applied. In control solutions, the vehicle was diluted in the same concentration as in the solutions containing drugs. Bovine serum albumin (BSA) was added in a concentration of 0.1 mg/ml to the solutions used for experiments with WIN 55,212-3.

## 3 Results

**Fig. 1:**
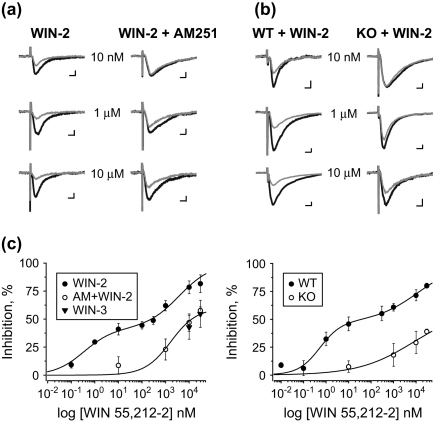
The suppression of excitatory postsynaptic currents by WIN 55,212-2 via CB_1_R-dependent and independent mechanisms in rats and mice. **a**, In rat slices, representative averaged records of 6–12 consecutive events taken before (black) and after 10 min of WIN 55,212-2 application (gray) in the absence or presence of 2 μM AM251 are superimposed. **b**, Averaged recordings of 8–12 consecutive EPSCs taken before (black) and after application of WIN 55,212-2 (WIN-2; gray) in wild type mice (WT) or in CB_1_R knockouts (KO). The concentration of the CB_1_R agonist is indicated for each example. Scale bars are 25 pA and 5 ms. **c**, Concentration-response relationship of WIN 55,212-2 in the inhibition of evoked EPSCs recorded in CA1 pyramidal cells in rats (left panel) and mice (right panel). The agonist inhibited the amplitude of events in two steps in rats and wild type mice, whereas only high doses of WIN 55,212-2 reduced synaptic currents in the presence of AM251 or in CB_1_R knockout mice. Data obtained after application of the inactive enantiomer WIN 55,212-3 (WIN-3) are also included on the left graph.

The effects of the cannabinoid agonist WIN 55,212-2 on EPSCs evoked by focal stimulation of Schaffer collaterals were measured in hippocampal CA1 pyramidal cells. First we performed concentration response analyses for the inhibitory effects of WIN 55,212-2 on evoked EPSC in rat slices (Fig. 1a). WIN 55,212-2 bath applied between the concentrations of 0.1 nM and 30 μM suppressed the amplitude of EPSCs in two steps. The apparent EC_50_ values from the fitted curve were 2.91 nM and 3.77 μM (Fig. 1c). Then we investigated the WIN 55,212-2-sensitivity of EPSCs, when AM251, a CB_1_R specific antagonist was added to the bath solution in the concentration of 2 μM. In spite of the presence of AM251, the cannabinoid agonist could still reduce the amplitude of evoked currents, but only in the μM range (Fig. 1a). The estimated EC_50_ value for this effect was 1.69 μM (Fig. 1c).

In the next set of experiments, we examined the concentration response relationship for the WIN 55,212-2-induced reduction of evoked EPSCs in mouse slices (Fig. 1b). The sensitivity of synaptic currents for WIN 55,212-2 was tested between the concentrations of 0.01 nM and 30 μM. Similar to that observed in rat slices, the cannabinoid agonist also decreased the amplitude of EPSCs in two steps. The EC_50_ values estimated by fitting a curve to the points of the dose response plot were 1.91 nM and 12.1 μM (Fig. 1c). To reveal whether WIN 55,212-2 could still suppress excitatory transmission in CB_1_R-independent manner in mice, we examined the effect of the cannabinoid agonist in CB_1_R knockout animals. As shown in Fig. 1b, WIN 55,212-2 effectively reduced the amplitude of EPSCs, but only in the μM range. The apparent EC_50_ value estimated from the curve fitting was 8.32 μM (Fig. 1c).

These results obtained both in rats and mice suggest that WIN 55,212-2 in nM concentrations inhibits excitatory synaptic transmission exclusively via CB_1_Rs, whereas in μM concentrations it has a mixed CB_1_R-dependent and -independent effect on glutamatergic transmission at Schaffer collateral synapses.

**Fig. 2:**
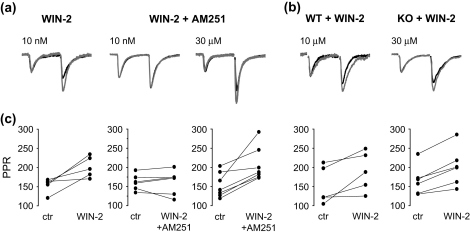
Both CB_1_R-dependent and -independent effects of WIN 55,212-2 enhance the paired-pulse facilitation of evoked EPSCs. **a**, In rat slices, the paired-pulse ratio was similarly increased after application of WIN 55,212-2 (WIN-2) in low concentrations or in high concentrations, when 2 μM AM251 was present in the bath, as seen on the scaled representative averages of 10–12 consecutive events before (black) and after (gray) the drug treatments. However, the paired pulse ratio remained unchanged, when WIN 55,212-2 in 10 nM concentration was co-applied with AM 251. **b**, Averaged recordings of consecutive EPSCs taken before (black) and after application of WIN 55,212-2 (gray) in wild type mice (WT) or in CB_1_R knockouts (KO) were scaled to indicate the enhancement of the paired-pulse ratio. The stimulus artefacts were removed from the traces. **c**, The paired-pulse ratios (PPR) calculated from each recordings in control conditions (ctr) and after drug application are presented for corresponding experiments.

By a comparison of the paired-pulse ratios of evoked EPSCs, we next investigated whether the CB_1_R-independent action of WIN 55,212-2 is presynaptic, i.e. whether it is inhibiting glutamate release similar to that seen earlier for CB_1_Rs. We first examined the effect of 10 nM WIN 55,212-2 on the paired-pulse ratio in rats and wild type mice. After drug application, the ratio significantly increased to 132.5 ± 9.4% of control in rats and to 129.5 ± 14.2% of control and mice (Fig. 2; *n* = 5 each, *p* < 0.05, Wilcoxon test). These data are in line with both electrophysiological and anatomical results, suggesting a presynaptic locus of CB_1_R-dependent action. To check that the changes in the paired-pulse ratio were due to the activation of CB_1_Rs, we contrasted these values before and after the application of 10 nM WIN 55,212-2 in the presence of AM251. As expected, the paired-pulse ratio was not altered (98.4 ± 6.1%, *n* = 6; *p* > 0.1, Wilcoxon test; Figs. 2a,c). Next we compared the paired-pulse ratio before and after the application of 30 μM WIN 55,212-2. The ratio of evoked currents was significantly increased to 145.1 ± 5.3% of control in rat slices (*n* = 5; *p* < 0.05, Wilcoxon test) and to 141.3 ± 11.8% in slices from wild type mice (*n* = 7; *p* < 0.05, Wilcoxon test). To reveal that the CB_1_R-independent action of WIN 55,212-2 also modifies transmitter release, we investigated the paired-pulse ratio after application of 30 μM WIN 55,212-2, while 2 μM AM251 was included in the bath. The ratio of the amplitude of evoked EPSCs still significantly increased, to 136.4 ± 8.4% of control (Figs. 2a,c; *n* = 7, *p* < 0.05, Wilcoxon test). Similar to these results, 30 μM WIN 55,212-2 also raised the paired pulse ratio to 121.1 ± 2.9% of control in CB_1_R knockout mice (Figs. 2b,c; *n* = 6, *p* < 0.05, Wilcoxon test). Thus, the CB_1_R-independent effect of WIN 55,212-2 also appears to be presynaptic, reducing glutamate release from Schaffer collateral terminals.

As reported earlier (Shen and Thayer, 1998), WIN 55,212-2 in μM concentrations could directly alter Ca^2+^ currents independent of CB_1_Rs, an effect that could be mimicked by its inactive enantiomer WIN 55,212-3. To test whether at glutamatergic axon terminals a similar mechanism would be responsible for the reduction of EPSC amplitude, WIN 55,212-3 was bath applied in two different concentrations to rat slices. This inactive enantiomer significantly suppressed the amplitude of evoked EPSCs by 42.8 ± 10.7% (*n* = 5) and 54.7 ± 12.1% (*n* = 3) in 10 μM and 30 μM concentrations, respectively (Fig. 1c). These effects were indistinguishable from those values, which were obtained in the presence of AM251 after application of 10 μM (46.9 ± 7.8%; *n* = 5) or 30 μM (59.6 ± 3.2%; *n* = 4) WIN 55,212-2 (*p* > 0.1, Mann–Whitney *U*-test). These results suggest that the CB_1_R-independent action of WIN 55,212-2 on glutamatergic transmission might be due to the direct inhibition of Ca^2+^ entry into the presynaptic boutons.

**Fig. 3:**
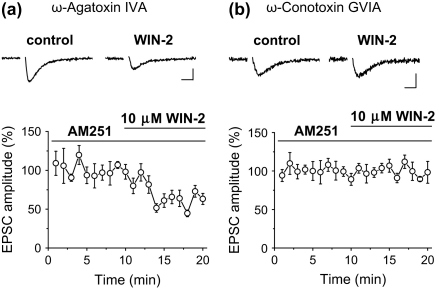
CB_1_R-independent effect of WIN 55,212-2 at excitatory synapses is mediated via inhibition of N-type Ca^2+^ channels. Rat slices were pre-treated with 250 nM ω-agatoxin IVA or with ω-conotoxin GVIA at least for an hour before the experiments. To block CB_1_Rs, 2 μM AM251 was included in the solution. **a**, In slices pre-incubated with ω-agatoxin IVA, 10 μM WIN 55,212-2 effectively reduced the amplitude of evoked EPSCs as shown on the averaged recordings of 8–10 consecutive events before (right) and after (left) drug application. The bottom graph calculated from 4 experiments indicates that wash-in of 10 μM WIN 55,212-2 significantly suppressed the EPSC amplitude. **b**, In contrast, when 10 μM WIN 55,212-2 was applied onto slices that were pre-incubated in ω-conotoxin GVIA, no change in the amplitude of EPSC was observed. Averaged traces before (right) and after (left) drug application are shown. The stimulus artefacts were removed from the traces. Scale bars are 20 pA and 5 ms. The bottom plot obtained from 6 experiments shows that WIN 55,212-2 could not alter the glutamatergic transmission, indicating that, independent of CB_1_Rs, N-type voltage-gated Ca^2+^ channels are required for presynaptic inhibition by this cannabinoid compound applied in high doses.

To get deeper insight into the mechanisms underlying the CB_1_R-independent effects of WIN 55,212-2, we specifically examined the involvement of voltage-gated Ca^2+^ channels in this process. Rat slices were pre-incubated either in 250 nM ω-agatoxin IVA (a specific blocker of P/Q-type Ca^2+^ channels) or in 250 nM ω-conotoxin GVIA (a specific inhibitor of N-type Ca^2+^ channels) at least for an hour. After placing the pre-treated slices in the recording chamber, we bath applied 10 μM WIN 55,212-2 in the presence of 2 μM AM251. In slices pre-treated with ω-agatoxin IVA, the amplitude of EPSCs was reduced by 39.7 ± 8.3% (*n* = 4, *p* < 0.05, Wilcoxon test; Fig. 3a), whereas there was no change in the synaptic currents after application of WIN 55,212-2 in slices pre-incubated with ω-conotoxin GVIA (95.8 ± 3.2% of control, *n* = 6, *p* > 0.1, Wilcoxon test; Fig. 3b). The results of these experiments suggest that the CB_1_R-independent action of WIN 55,212-2 at glutamatergic synapses is mediated via inhibition of N-type Ca^2+^ channels.

## 4 Discussion

Our data presented here demonstrate that excitatory synapses of Schaffer collaterals in CA1 pyramidal cells are inhibited by WIN 55,212-2 both via CB_1_R-dependent and independent mechanisms. In low nM concentrations, this cannabinoid ligand solely acts as a CB_1_R agonist reducing glutamate release. In contrast, WIN 55,212-2 in the μM range suppresses glutamatergic synaptic transmission via activation of CB_1_Rs as well as inhibiting N-type Ca^2+^ channels independent of CB_1_Rs.

Shen et al., (1996) were the first to show that excitatory transmission in the hippocampus could be reduced by WIN 55,212-2, a finding that has been strengthened later by several other laboratories (Misner and Sullivan, 1999; Al-Hayani and Davies, 2000; Hájos and Freund, 2002; Ohno-Shosaku et al., 2002; Hoffman et al., 2003). Similarly to that observed in the hippocampus, WIN 55,212-2 was also shown to suppress excitatory synapses in other brain regions, including the cerebellum (Levenes et al., 1998; Takahashi and Linden, 2000), neocortex (Domenici et al., 2006), basolateral amygdala (Azad et al., 2003; Domenici et al., 2006), or striatum (Gerdeman and Lovinger, 2001; Huang et al., 2001). In earlier studies, the lack of immunostaining for CB_1_Rs at excitatory terminals (Katona et al., 1999; Hájos et al., 2000), taken together with experiments showing that WIN 55,212-2 could significantly reduce glutamate release in CB_1_R knockouts (Hájos et al., 2001; Kofalvi et al., 2003), fuelled the concept that distinct cannabinoid receptors control synaptic excitation and inhibition. This interpretation was supported by the unequivocal demonstration of high densities of CB_1_Rs on GABAergic axons, while adjacent glutamatergic terminals remained negative (Katona et al., 1999; Hájos et al., 2000; Nyiri et al., 2005), as well as by the complete disappearance of cannabinoid sensitivity of IPSCs in CB_1_R knockouts (Hájos et al., 2000, 2001; Varma et al., 2001; Wilson et al., 2001). Moreover, Hájos and Freund (2002) showed that AM251 could fully antagonise the effect of WIN 55,212-2 at GABAergic, but not at glutamatergic synapses, further strengthening the existence of a novel cannabinoid-sensitive binding site at hippocampal excitatory synapses. Recently this concept was substantially challenged both by anatomical and electrophysiological experiments, including studies from our own laboratory. First, using a different type of antibody, CB_1_Rs were convincingly shown to be present on glutamatergic terminals, although in much smaller quantities than on GABAergic axons (Katona et al., 2006; Kawamura et al., 2006). Specificity of the staining has been confirmed in CB_1_R knockout tissue. Second, excitatory transmission was found to be insensitive to the application of WIN 55,212-2 in distinct strains of transgenic mice lacking CB_1_Rs (Ohno-Shosaku et al., 2002; Domenici et al., 2006; Takahashi and Castillo, 2006). The discrepancy between earlier and recent data might be resolved by the present findings suggesting that at low nM concentrations WIN 55,212-2 specifically activates CB_1_Rs, whereas in the μM range the agonist could further reduce glutamate release via direct inhibition of presynaptic Ca^2+^ entry independent of CB_1_Rs (present study; Shen and Thayer, 1998; Kofalvi et al., 2007). This difference in the specificity of WIN 55,212-2 as a function of its concentration can be noticed already in studies reported by the Kano laboratory. WIN 55,212-2 in 100 nM caused a large reduction in the amplitude of EPSCs in wild type mice, but in CB_1_R knockouts less then 5% suppression was found (Ohno-Shosaku et al., 2002). However, in 2 μM concentration, WIN 55,212-2 inhibited excitatory transmission by about 20% in CB_1_R knockout mice, an effect that was unaltered in the presence of CB_1_R antagonists (Kawamura et al., 2006). Further support to the hypothesis that WIN 55,212-2 in μM concentrations can reduce excitatory transmission via CB_1_R-independent mechanisms come from the work of Hoffman et al. (2005), where the authors showed that in the presence of AM251, 3 μM WIN 55,212-2 significantly (appr. by 40%) reduced excitation. This view, however, is not supported by two recent studies using CB_1_R knockout animals, in which 5 μM WIN 55,212-2 was found to be completely ineffective at excitatory synapses (Domenici et al., 2006; Takahashi and Castillo, 2006). The explanation for these negative findings remains to be investigated.

Another finding of the present study that deserves discussion is that the effective concentration of WIN 55,212-2 that significantly inhibited the amplitude of synaptic events in slice preparations was 100 fold lower than it was earlier reported by several laboratories using similar recording circumstances (Takahashi and Linden, 2000; Robbe et al., 2001; Hájos and Freund, 2002; Hoffman et al., 2005). Compared to earlier studies, we changed some conditions that could account for the distinct efficacy of WIN 55,212-2, which allowed clearly separating CB_1_R-dependent and independent effects. The preparation and storage of slices, as well as the flow rate of the solution during recordings was modified: slices were cut in a sucrose containing solution and stored in an interface-type chamber before recordings, and a higher flow rate was used in the recording chamber, ensuring a better oxygenation of the tissue (Hájos et al., 2005). Under these circumstances, the amplitude of evoked synaptic currents became more stable, and, more importantly, the efficacy of WIN 55,212-2 to suppress excitatory synapses was comparable with those measured in cell cultures (Ohno-Shosaku et al., 2002) or binding assays (Felder et al., 1995).

Several studies in different brain regions suggested that the CB_1_R-independent effect of WIN 55,212-2 might significantly alter synaptic communication among neurons (Hájos et al., 2001; Pistis et al., 2004; Kofalvi et al., 2005; Matyas et al., 2006), presumably through a direct blockade of Ca^2+^ entry at the presynaptic terminals (Shen and Thayer, 1998; Kofalvi et al., 2007). In the present study, we provided evidence that the CB_1_R-independent effect of WIN 55,212-2 at glutamatergic synapses was mediated by inhibiting N-type Ca^2+^ channels.

The question arises whether WIN 55,212-2 in high doses could also alter GABAergic transmission independent of CB_1_Rs, since GABA release from CB_1_R-expressing axon terminals is known to depend on N-type Ca^2+^ channel activation (Wilson et al., 2001). Indeed, we found that in CB_1_R knockout mice 10 μM WIN 55,212-2 substantially reduced the amplitude of IPSCs to 62.8 ± 20.5% of control (*n* = 4). In contrast, when CB_1_R function was intact, both low and high doses of WIN 55,212-2 led to a comparable reduction of IPSC amplitudes (in 3 nM: 47.9 ± 15.4% of control, *n* = 4; in 10 μM: 45.2 ± 19.7% of control, *n* = 4), similar to results obtained earlier (Hájos and Freund, 2002). The reason why WIN 55,212-2 in 10 μM concentration did not result in an additional suppression of inhibitory events in the presence of functional CB_1_Rs may be explained by the fact that CB_1_R-dependent inhibition of GABAergic currents is entirely due to blocking N-type Ca^2+^ channels, which occludes the CB_1_R -independent action of WIN 55,212-2 directly on the same Ca^2+^ channels. Testing this hypothesis, and other novel aspects of cannabinoid modulation of GABAergic transmission is the subject of another line of investigations in our laboratory.

The question arises whether at glutamatergic synapses, under some experimental conditions, endocannabinoids can reduce the efficacy of neurotransmission via CB_1_R-dependent and -independent mechanisms, similar to high concentrations of WIN 55,212-2, which can modify Ca^2+^ entry directly. Some results indeed imply that endocannabinoids could directly inhibit different types of voltage-gated Ca^2+^ channels independent of CB_1_Rs (Chemin et al., 2001; Fisyunov et al., 2006), yet other data suggest that endocannabinoids released upon depolarization of a postsynaptic neuron (or exogenously applied) are unable to suppress excitatory transmission in CB_1_R knockout mice (Ohno-Shosaku et al., 2002; Straiker and Mackie, 2005, but see Rouach and Nicoll, 2003). Nevertheless, the importance of identifying a CB_1_R-independent binding site for WIN 55,212-2 as N-type Ca^2+^ channels at excitatory terminals lies in resolving some contradictions in pharmacological and behavioural studies that emerged partly due to the use of WIN 55,212-2 in widely varying concentrations (see a thorough discussion of this issue in Haller et al., 2007).
